# 3D-Flower-Like Copper Sulfide Nanoflake-Decorated Carbon Nanofragments-Modified Glassy Carbon Electrodes for Simultaneous Electrocatalytic Sensing of Co-existing Hydroquinone and Catechol

**DOI:** 10.3390/s19102289

**Published:** 2019-05-17

**Authors:** Lina Abdullah Alshahrani, Liqiong Miao, Yanyu Zhang, Shengming Cheng, Palanivel Sathishkumar, Balasubramaniam Saravanakumar, Junmin Nan, Feng Long Gu

**Affiliations:** Key Laboratory of Theoretical Chemistry of Environment, Ministry of Education; School of Chemistry and Environment, South China Normal University, Guangzhou 510006, China; lamy7667@hotmail.com (L.A.A.); 20162421064@m.scnu.edu.cn (L.M.); 15521435886@163.com (Y.Z.); raindyjohnson@163.com (S.C.); salemsathishkumar@gmail.com (P.S.); mbsaravanakumar@gmail.com (B.S.); jmnan@scnu.edu.cn (J.N.)

**Keywords:** carbon nanofragments, copper sulfide, catechol, hydroquinone, electrochemical sensor, simultaneous determination

## Abstract

A copper sulfide nanoflakes-decorated carbon nanofragments-modified glassy carbon electrode (CuS-CNF/GCE) was fabricated for the electrocatalytic differentiation and determination of hydroquinone (HQ) and catechol (CC). The physicochemical properties of the CuS-CNF were characterized by scanning electron microscopy, transmission electron microscopy, X-ray diffraction, X-ray photoelectron spectroscopy and Raman spectroscopy. The electrocatalytic determination of HQ and CC over the CuS-CNF/GCE was evaluated by cyclic voltammetry and differential pulse voltammetry. An excellent detection limit and sensitivity of the CuS-CNF/GCE are obtained (0.293 µM and 0.259 µM) with a sensitivity of 184 nA µM^−1^ cm^−2^ and 208 nA µM^−1^ cm^−2^ (S/N=3) for HQ and CC, respectively. In addition, the CuS-CNF/GCE shows a selective identification of HQ and CC over potential interfering metal ions (Zn^2+^, Na^+^, K^+^, NO^3−^, SO_4_^2−^, Cl^−^) and organic compounds (ascorbic acid, glucose), and a satisfactory recovery is also obtained in the spiked water samples. These results suggest that the CuS-CNF/GCE can be used as an efficient electrochemical sensor for the simultaneous determination of co-existing environmental pollutants such as HQ and CC in water environments with high selectivity and acceptable reproducibility.

## 1. Introduction

Dihydroxybenzene isomers such as hydroquinone (HQ) and catechol (CC) have been extensively used in the cosmetics, dye, pharmaceutical, plastics and pesticide manufacturing industries [1,2]. During industrial process, HQ and CC are generally released into the aqueous environment, which can cause serious environmental impacts due to their high genotoxicity and carcinogenicity, even at low concentrations. Thus, the European Union and the United States Environmental Protection Agency have categorized these isomers as harmful pollutants [3,4]. The rapid and accurate detection of these isomers in the aqueous environment is a prerequisite for their effective management and treatment, but in practice, it is difficult to distinguish HQ and CC in environmental samples because of their similar chemical structure and properties [5]. So far, although several methods including chromatography [6], electrochemical sensors [7,8], spectrophotometry [9] and fluorescence [10] have been developed for the determination of dihydroxybenzene isomers, simple, cost-effective, selective and sensitive techniques are still needed.

As a promising method, electrochemical sensors are highly attractive for the simultaneous determination of dihydroxybenzene isomers because of their advantages like inexpensive instrumentation, rapid evaluation and high sensitivity [11,12]. However, the overlapping oxidation and reduction peaks of HQ and CC on bare electrodes hinder their simultaneous determination [13]. Therefore, the exploration of functional materials-modified electrodes to extend the dynamic range of analytical determinations still remains a major challenge for the electrochemical determination of HQ and CC. So far, various carbon-based nanomaterials such as carbon nanocages (CNCs) [14], carbon nanofibers [15], carbon nanotubes (CNTs) [16], carbon nanofragments (CNFs) [17], carbon dots (CDs) [18], graphene-like carbon nanosheets (GCN) [19] and activated graphene oxide [20] have been developed for the electrochemical detection of HQ and CC. Thereinto, CNFs reveal unique nanostructured fragments with high surface area, abundant functional groups, excellent structural stability and high electrical conductivity and electrocatalytic oxidation efficiency [21]. Interestingly, the CNFs derived from spent graphite exhibit various applications after converting into another form [17,22]. Our previous work confirmed the efficacy, selectivity and electrocatalytic performance of metal-based nanomaterials-anchored CNFs for the simultaneous determination of HQ and CC [22]. Also, the study indicated that the preparation of the metal-based nanomaterials-anchored CNF may be an effective method to improve the performance of the modified electrode. 

It is noted that as a binary functional material, copper sulfides usually exist in the form of CuS (covellite) and Cu_2_S (chalcocite) [23]. In particular, CuS nanoparticles are widely used in solar cells, sensors and batteries due to their non-linear optical properties, high conductivity, excellent solar radiation absorbing properties and environmental compatibility [24,25]. Interestingly, a CuS nanotubes-modified electrode showed an excellent electrochemical sensing of glucose in an enzyme-free environment [26]. Inspired by the good electrochemical sensing properties of CuS, it is expected that a CuS-decorated CNF may be an effective electrode modifier. In this work, CuS nanoflakes-decorated CNFs (CuS-CNF) were synthesized by a controllable refluxing method, and then the CuS-CNF used to modify glassy carbon electrode (CuS-CNF/GCE) for simultaneous determination of CC and HQ. The fabricated CuS-CNF/GCE showed high sensitivity, selectivity and reliability for the simultaneous determination of CC and HQ in environmental water samples.

## 2. Experimental

### 2.1. Chemicals

HQ, CC and N,N-dimethylformamide (DMF) were purchased from Sinopharm Chemical Reagent Co., Ltd. (Shanghai, China). The Li-ion batteries used were obtained from Guangzhou Tinci Materials Technology Co., Ltd. (Guangdong, China). Polyvinylpyrrolidone (PVP), ethylene glycol, copper chloride dehydrate (CuCl_2_.2H_2_O), thiourea (CH_4_N_2_S), zinc sulfate monohydrate (ZnSO_4_.H_2_O), potassium chloride (KCl), sodium nitrate (NaNO_3_), ascorbic acid (C_6_H_8_O_6_) and D-glucose (C_6_H_12_O_6_) were obtained from Aladdin Chemicals (Shanghai, China). All solutions were prepared using ultrapure water (18.2 MΩ·cm) from a Milli-Q system (Millipore, Bedford, MA, USA). Phosphate buffer solution (PBS; 0.1 mol L^−1^) with different pH values (3–8) were prepared by using NaH_2_PO_4_ and Na_2_HPO_4_. 

### 2.2. Preparation of the CuS-CNF/GCE

The CNF was prepared from spent graphite which recovered from cycled Li-ion batteries according to the protocol described in our previous work [27]. For the synthesis of CuS-CNF, PVP (1 g) was dissolved in ultrapure water (40 mL), followed by addition to a solution of CuCl_2_ (2 mmol) and thiourea (4 mmol) dissolved in ethylene glycol (30 mL). Further, the prepared solution was mixed with well dispersed CNF (20 mg of CNF in 50 mL of DMF) and refluxed at 160 °C for 2 h. The obtained precipitate was repeatedly washed with ultrapure water and ethanol through centrifugation (8000 rpm; 5 min). The collected pellet was dried in a vacuum oven at 60 °C for 12 h. Then, 2 mg of CuS-CNF was dispersed in 1 mL of ultrapure water and used for electrode modification. Before modifying the GCE with CuS-CNF, the GCE was polished with 0.3 and 0.03 µm alumina slurry and washed with ultrapure water and ethanol, and finally dried by N_2_ gas. Then, 10 µL of CuS-CNF suspension was dropped on the GCE surface and air-dried under ambient conditions. Similarly, CNF modified electrode was also prepared using the same procedure. 

### 2.3. Apparatus and Characterization

The electrochemical detection of HQ and CC were tested by cyclic voltammetry (CV) and differential pulse voltammetry (DPV) using CHI660B electrochemical workstation (Chenhua, China). In the three-electrode system, Ag/AgCl (3 mol L^−1^ KCl), Pt foil and modified GC were used as a reference, counter and working electrodes, respectively. DPV was measured in the potential window of −0.2 – 0.6 V, with a pulse width of 0.25 s, an increment of 0.005 V, a pulse period of 0.5 s and a voltage amplitude of 0.05 V. The surface morphology of the CNF and CuS-CNF were analyzed by scanning electron microscopy (SEM, ZEISS Ultra-55, Carl Zeiss, Oberkochen, Germany) and transmission electron microscopy (TEM, JEM-2100, JEOL Ltd., Tokyo, Japan). The structural and chemical states of the materials were analyzed using X-ray diffraction pattern (XRD, Bruker, Karlsruhe, Germany) and X-ray photoelectron spectroscopy (XPS, ESCALAB 250Xi, Thermo Scientific, Waltham, MA, USA). The pH was measured using a PHSJ-3F pH instrument (Leici, Shanghai, China). Raman measurements were carried out in Alpha 300R instrument (WITec GmbH, Ulm, Germany) at a wavelength of 532 nm. The electrochemical impedance was measured on a CHI660B system (CH Instruments, Austin, TX, USA) at the frequency range of 0.1 to 100 kHz with an amplitude of 10 mV.

## 3. Results and Discussion

### 3.1. Characterization of the CuS-CNF Nanomaterials

**Figure 1:**
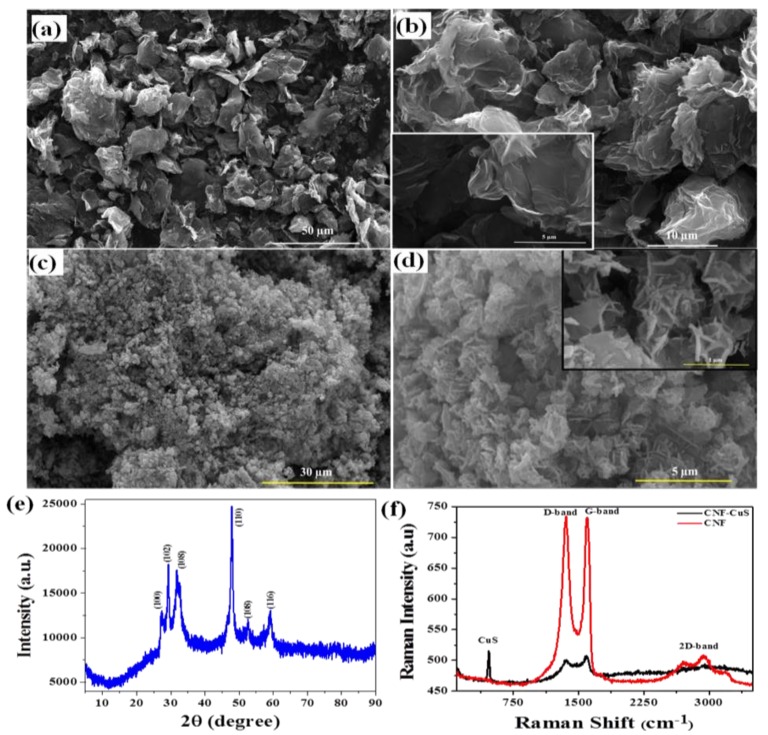
SEM images of (**a**,**b**) CNF (inset: higher magnified image) and (**c**,**d**) CuS-CNF; (**e**) XRD pattern CuS-CNF; and (**f**) Raman Spectra of bare CNF and CuS-CNF.

The surface morphology and structural characteristics of the as-prepared CNF and CuS-CNF were analyzed through SEM, XRD and Raman measurements. A wrinkled thin sheet-like structure can be seen in the SEM images of the CNF (Figure 1a,b). 

The SEM images of CuS-CNF shown in Figure 1c,d indicate the uniform and homogenous growth of CuS nanoflakes on CNF, and the flakes are self-assembled to form an interconnected 3D-flower like structure. In addition, XRD was also measured to find out the structural information of CuS-CNF, as shown in Figure 1e. Sharp diffraction peaks at 27.3, 29.5, 31.9, 47.8, 52.3 and 59.1 corresponding to the (100), (102), (103), (110), (108) and (116) planes of CuS with hexagonal structure (covellite, space group: P63/mmc(194)) (JCPDS No. 006-0464) can be seen [28]. There was no clear peak in the structure corresponding to the CNF, thus Raman measurements were carried out to confirm the presence of the CNF and to study the structural disorder in the CNF. As shown in Figure 1f, the Raman spectrum of the CNF clearly indicates two distinct peaks at 1358.7 and 1596.5 cm^−1^ corresponding to the structural deformation of the in-plane sp^2^ domain (D peak) and in-plane vibrational mode (G peak). Besides these peaks, an extra peak appeared at 2700–3000 cm^−1^ corresponding to the 2D peak. A higher I_D_/I_G_ value specifies the larger number of structural defects in the graphitic plane in the CNF. In CuS-CNF, an additional peak appeared at 474 cm^−1^, indicating the presence of CuS. For clear visualization, the TEM analysis of CuS-CNF and its corresponding elemental mapping were recorded. 

**Figure 2:**
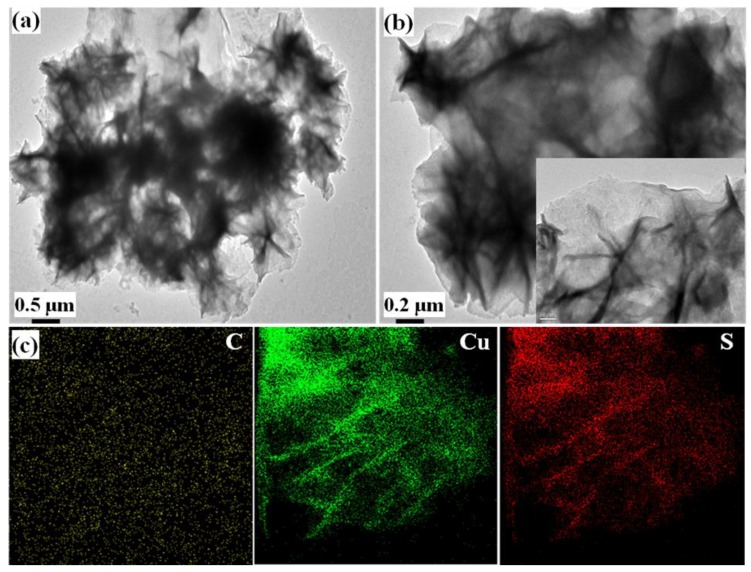
TEM images of (**a**,**b**) CuS-CNF and their (**c**) corresponding elemental maps.

Figure 2a,b demonstrate the homogeneous distribution of CuS on layered CNF sheets. In addition, the wrinkled nature of CNF sheets indicates the very small sheet thickness. Moreover, the elemental analysis also confirmed the uniform distribution of the Cu, S, and C elements in the CNF surface.

**Figure 3:**
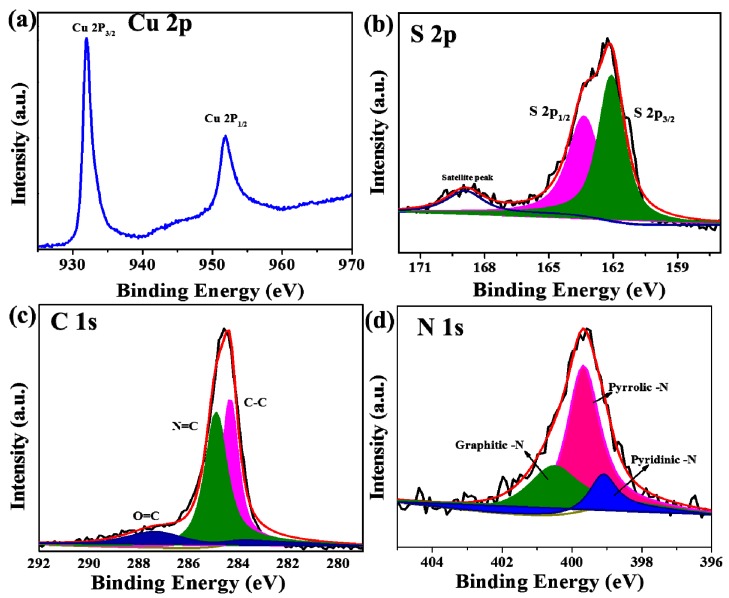
XPS spectra of CuS-CNF: (**a**) high resolution Cu 2p spectrum, (**b**) high resolution S 2p spectrum, (**c**) high resolution C 1s spectrum, and (**d**) high resolution N 1s spectrum.

The purity and chemical oxidation state were measured by XPS analysis, as shown in Figure 3 and Figure S1. The XPS survey spectrum of CuS-CNF indicates the presence of the Cu, S and O elements in the prepared sample (Figure S1). From the high-resolution Cu 2p spectrum (Figure 3a), the two broad peaks observed at 932 and 951.9 eV corresponds to the spin-orbit of Cu 2p_3/2_ and Cu 2p_1/2_, respectively. Furthermore, the peak separation of two spin-orbit was ~19.9 eV, which confirms the existence of Cu^1+^ ions in CuS and is comparable to the reported value [29,30]. Further, there is no satellite peak clearly indicating the Cu^1+^ oxidation state [31]. In a similar way, the high-resolution S 2p peak also has a two spin-orbit splitting of S 2p_3/2_ and S 2p_1/2_ at 162.1 and 163.4 eV with a peak separation of 2.3 eV clearly showing the presence of S^2−^ in the composite.

Moreover, an additional peak appeared at 168.9 eV corresponds to satellite peak. In high-resolution C 1s spectrum (Figure 3c), three peaks appeared at 284.3, 284.9 and 287.9 eV were represents the sp^3^/sp^2^ carbon, C-N and C-O, respectively. Furthermore, N 1s core-level spectrum (Figure 3d) consists of three peaks at 399.1, 399.6 and 400.6 eV corresponds to pyridine-N, pyrrolic-N and graphitic-N, respectively [32,33]. Further, the N doping enhances the electrochemical kinetics of the composite and N doping may occur during the refluxing of thiourea. 

### 3.2. Electrochemical Performances of the CuS-CNF/GCE

**Figure 4:**
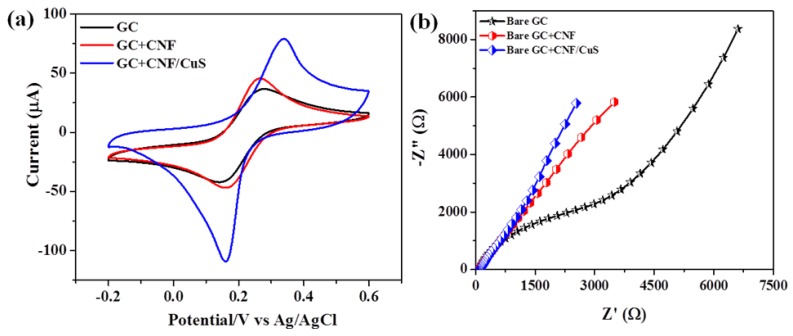
(**a**) CV curve at a scan rate of 50 mV s^−1^ and (**b**) EIS spectra of different electrodes in 1 mM [Fe(CN)_6_]^3−/4−^ in 0.1 M KCl solution.

The electrochemical performance of the modified electrodes was analyzed by CV and electrochemical impedance (EIS) measurement in the presences of 1 mM [Fe(CN)_6_]^3^^−/4−^ in 0.1 M KCl solution at a scan rate of 50 mV s^−1^ (Figure 4).

Figure 4a shows the CV curve of different electrodes. Interestingly, a higher redox current (Ip_a_ = 80 µA) was observed for CuS-CNF/GCE compared to bare GCE (Ip_a_ = 37.4 µA) and CNF/GCE (Ip_a_ = 46.7 µA), which may be due to the excellent catalytic properties of CuS and the conductive path provided by CNF. The charge transfer characteristics of the different electrodes were analyzed through EIS. As shown in Figure 4b, the result clearly indicates the lower charge transfer (R_CT_: 103 Ω) characteristics of the CuS-CNF/GCE compared to the other two electrodes. It is obvious that the fabricated CuS-CNF/GCE has good interfacial electron transfer properties and is very consistent with the CV results. 

**Figure 5:**
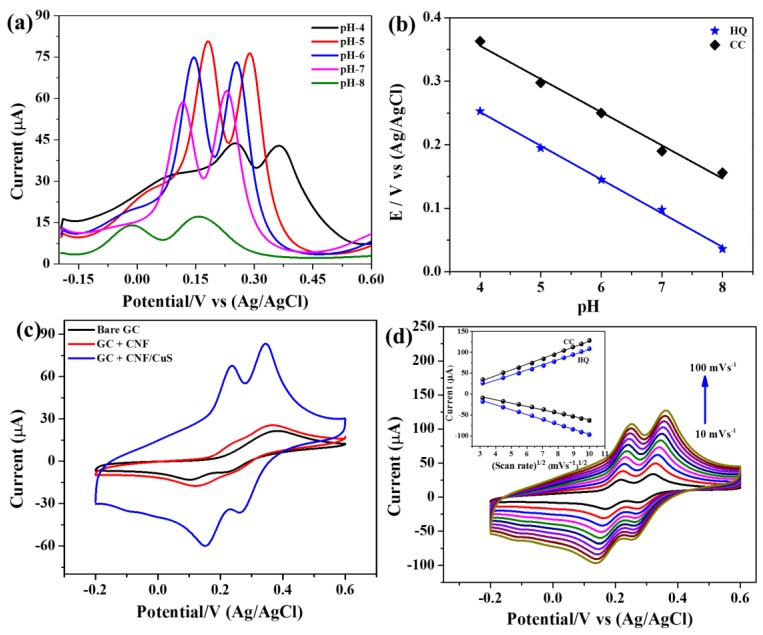
(**a**) DPV curves, (**b**) potential vs pH of CuS-CNF/GCE in 0.1 M PBS containing 50 µM of HQ and CC at different pH value. (**c**) CV curves of different electrodes at 50 mV s^−1^, and (**d**) CV curves of CuS-CNF/GCE in 0.1M PBS (pH = 5) containing a 50 µM of HQ and CC at different scan rates (inset: redox peak potential vs. square root of scan rate).

Furthermore, the electrochemical detection of the CuS-CNF/GCE was carried out in 0.1 M PBS at different pH values in the presence of HQ and CC (50 µM), and the results are shown in Figure 5a and Figure S2. The peak current has increased with increasing the pH from 3 to 5, and further, the increase of pH reduces the peak current (Figure S2). At the same time, a negative peak shift was observed with increasing pH (Figure 5b). At higher pH (>5), the peak current was decreased due to the higher electrostatic repulsion between the modified electrode and analytes. Hereafter, all the electrochemical measurements were carried out at pH 5. The regression equation of HQ and CC was calculated from Figure 5b, which was Ep(V) = −0.0531 pH + 0.464 (R = 0.9954) and Ep(V) = −0.0522 pH + 0.5646 (R = 0.9989) for HQ and CC, respectively. The slope of the regression equation represents the anode peak shift for each pH, and these values are in good agreement with the theoretical value (58.5 mV/pH) calculated according to the Nernst equation: $\left(dE_{p}/dpH\right)=-2.303mRT/nF$. This result concludes the overall electrochemical redox reaction was achieved based on two-electron and two-proton transfer process [34,35]. 

For comparison, CV curves were measured for all electrodes in 0.1 M PBS with 50 µM of HQ and CC at a scan rate of 50 mV s^−1^ (Figure 5c). The result shows a pair of redox peaks in the CV curves for all the electrodes, indicates the detection of HQ and CC in the electrolyte. However, a higher peak current was measured for CuS-CNF/GCE indicates the greater electrochemical kinetics of the electrode from CuS. Moreover, a small peak separation between the anodic and cathodic peaks of HQ and CC indicates the high electrochemical reversibility of the electrode, which is mainly due to the higher electrocatalytic property of CuS. The higher redox current and low over-potential of the CuS-CNF makes this material a good platform for the electrochemical detection of HQ and CC. The CuS-CNF/GCE was tested at different scan rates in 0.1 M PBS with 50 µM of HQ and CC at a scan rate of 50 mV s^−1^ (Figure 5d). The result shows that the anodic and cathodic peak currents of both HQ and CC were linearly increased with the increasing scan rates (Figure 5d inset). Moreover, the anodic and cathodic peaks shifted positive and negative sides, respectively, for increased the scan rates. The regression equation of HQ and CC indicates the electrochemical reaction followed a diffusion-controlled reaction. 

### 3.3. Electrochemical Detection of HQ and CC

**Figure 6:**
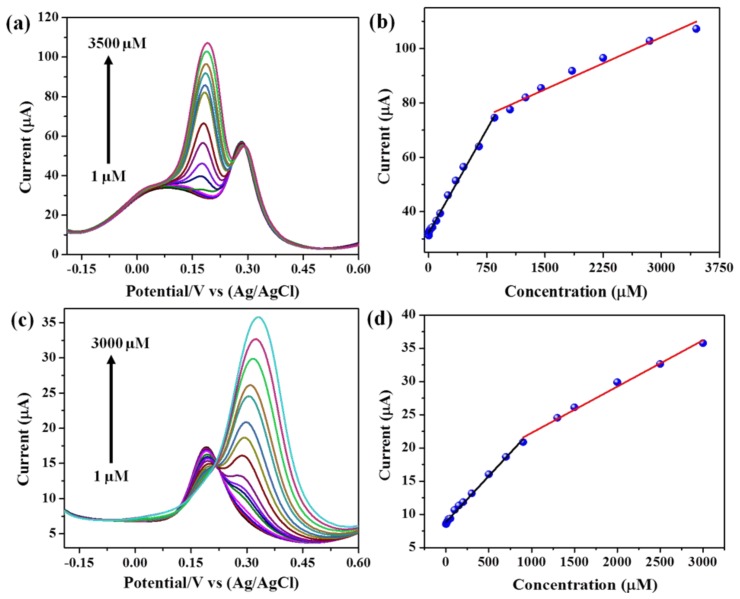
(**a**) DPV curves of CuS CuS-CNF/GCE in 0.1 M PBS (Ph = 5), (**a**) 50 µM of CC and different amount of HQ, (**b**) the current vs. the concentration of HQ, (**c**) 50 µM of HQ and different amount of CC, and (**d**) the current vs. the concentration of CC.

To check the selectivity of the modified electrode, we measured at one compound as a constant and varying the concentration of another compound through DPV measurement in the presence of 0.1 M PBS at pH 5. Figure 6a shows the DPV measurement at a constant CC (50 µM) with various amount of HQ from 1 to 3500 µM. While increasing the HQ concentration, the corresponding anodic current gradually increased and anodic peak current of CC maintained a constant value. Similarly, at increasing the CC concentration from 1 to 3000 µM, HQ maintained a constant current (Figure 6c). The regression equation of HQ was determined by linear fitting the anodic peak current vs HQ concentration plot. The resultant graph was plotted as shown in Figure 6b.

Two linear regions were found in the results; i.e, linear range: 1–850 and 850–3450 µM, which corresponds to the regression equations of I_HQ1_ = 31.79 + 0.0552 C_HQ_ (R = 0.998) and I_HQ2_ = 64.63 + 0.014 C_HQ_ (R = 0.995), respectively. Similarly, the linear region for different CC was calculated from Figure 6d, and the resultant linear region for CC was 1–900 µM; 900-3000 µM with corresponding regression equations of I_CC1_ = 8.846 + 0.0139 C_CC_ (R = 0.998); I_CC2_ = 16.28 + 0.0066 C_CC_ (R = 0.998), respectively. 

**Figure 7:**
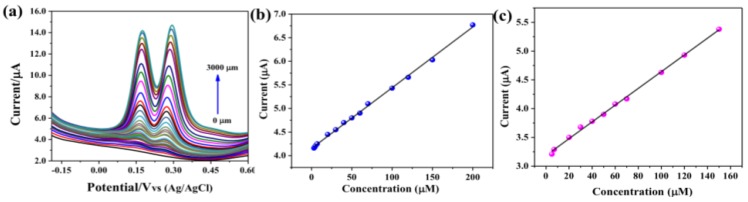
(**a**) DPV curves of CuS-CNF/GCE in 0.1 M PBS (pH = 5) containing different amounts of HQ and CC, (**b**) Current vs concentration of HQ and (**c**) Current vs concentration of CC.

The dihydroxybenzene isomers HQ and CC have similar chemical structures with higher current sensitivity, thus it is important to measure simultaneously. Figure 7a shows the simultaneous detection of HQ and CC in 0.1 M PBS from 1 to 3000 µM. There was a clear peak appeared for both HQ and CC component separately from low concentration to higher concentration, indicates the higher selectivity of the CuS-CNF/GCE. Further, the regression equation derived from the linear fitting of the peak current vs. concentration plot is shown in Figure 7b,c. The linear range for HQ and CC at simultaneous measurement were 3–200 µM and 7–150 µM and the corresponding regression equation was I_HQ_ = 4.15 + 0.0129 C_HQ_ (R = 0.999); I_CC_ = 3.187 + 0.0146 C_CC_ (R = 0.999), respectively. The calculated LOD of HQ and CC were 0.293 µM and 0.259 µM with a sensitivity of 184 nA µM^−1^ cm^−2^ and 208 nA µM^−1^ cm^−2^ (S/N=3), respectively. These results confirm that CuS-CNF is a good candidature for the simultaneous detection of co-existing dihydroxybenzene isomers such as HQ and CC with higher sensitivity and selectively. 

The performances of the CuS-CNF/GCE were better and competitive with previously reported values of metal-doped carbon materials modified GCE (Table 1). This high efficiency may due to the excellent physiochemical properties of the CuS present in CuS-CNF/GCE composite, which delivered a high sensing performance during electrochemical reaction [24,25]. Although the performance of the electrode material fabricated in this study is no better than that of a few other metal-based sensors, here we have successfully recycled waste battery materials for a useful waste-to-sensor” application. Notably, the CuS used in this study is also environmentally compatible [25]. Further, the performance of the electrode will be improved by controlled extraction of spent graphite from dead batteries. 

### 3.4. Interferences and Application of CuS-CNF/GCE

The interference studies were carried out in the presence of various metal ions (Zn^2+^, Na^+^, K^+^, NO^3−^, SO_4_^2−^, Cl^−^) and organic compounds (ascorbic acid, glucose) in the electrolyte (Figure S3). No significant changes in the detection of HQ and CC in this environment indicate the higher selectivity of the modified electrode towards HQ and CC. The repeatability of the CuS-CNF/GCE was tested under 25 repeated CV measurements in the presence of 50 µM HQ and CC in 0.1 M PBS. The standard deviations of the peak currents of HQ and CC were 3.1% and 3.5%, respectively, indicates excellent stability of CuS-CNF/GCE. Furthermore, in order to check the reproducibility of the proposed sensor, the experiment was performed with different CuS-CNF/GCE (5 nos.) at a similar condition. The result clearly shows only 2.5 and 3.2% deviation in the peak current for HQ and CC, respectively, confirms the higher reproducibility of the modified electrodes. 

The electrochemical sensing ability of CuS-CNF/GCE for real samples was tested in tap water. Since the concentrations of HQ and CC were very low in the tap water, no peak was observed. In order to detect HQ and CC in a real sample, we added different concentrations of HQ and CC to the tap water by the standard addition method and resultant recoveries of HQ and CC were 99–102% and 101–103%, respectively (Table 2). These results show the potential for practical application of CuS-CNF modified electrode.

## 4. Conclusions

In this work, we have successfully prepared CuS nanoflakes-anchored CNF by a controllable refluxing method and then used it to modify a GCE to detect HQ and CC. The results demonstrate that the electrochemical sensor properties of CNF derived from spend graphite (dead batteries) have been successfully improved by the combination of environmentally suitable CuS for simultaneous detection of co-existing pollutants with similar structures. In this CuS-CNF complex, novel and unique 3D-flower-like CuS nanoflakes provide higher electroactive sites for oxidizing HQ and CC; further, CNF provides a good conduction path for electrons during electrochemical reactions. The CuS-CNF-modified electrode showed higher selectivity, wide linear range, low LOD and excellent stability for HQ and CC detection. The proposed CuS-CNF/GCE is relatively simple to fabricate and has high reliability, and can simultaneously detect co-existing environmental pollutants HQ and CC. Furthermore, this novel “waste-to-sensor” concept may stimulate more interest in CNF for various electrochemical sensor applications.

## Figures and Tables

**Table 1:** Performance comparison of CuS-CNF/GCE with previously reported metal doped carbon materials modified GCE.

Metal Doped Carbon Materials Modified GCE	Linear range (µM)		LOD (µM)		References
	HQ	CC	HQ	CC	
Boron doped graphene	5–100	5–200	0.3	0.2	[36]
Fe/PC	0.1–120	1–120	0.014	0.033	[37]
NiO/CNT	10–500	10–400	2.5	2.5	[16]
NiO/MWCNT	7.4–56	7.4–56	0.039	0.015	[38]
GO-MnO_2_	0.01–0.7	0.03–1	0.007	0.01	[39]
CuO-CNF	3–80	0–150	1	2	[22]
Bi-CNF	-	3–20	-	0.2	[40]
CuS-CNF	3-200	7–150	0.293	0.259	This work

PC: porous carbon; CNT: carbon nanotube; GO: graphene oxide; CNF: carbon nanofragment; CuO: copper oxide; Bi: bismuth oxide.

**Table 2:** Detection of HQ and CC in tape water using CuS-CNF/GCE.

S. No.	Add (µM)		Found (µM)		Recovery (%)	
	HQ	CC	HQ	CC	HQ	CC
1	50	50	49.7	51.7	99.4	103.4
2	100	100	103.8	101.5	103.8	101.5
3	150	150	153.1	152	102.1	101.3

## Supplementary Materials

The following are available online at https://www.mdpi.com/1424-8220/19/10/2289/s1, Figure S1: XPS survey spectrum of CuS nanoflakes anchored CNF. Figure S2: a) CV curves and b) Current vs pH (DPV curve) of CuS-CNF/GCE in 0.1 M PBS containing 50 µM of HQ and CC at different pH value. Figure S3: Interference study of CuS-CNF/GCE in 0.1 M PBS containing various metal ions (Zn^2+^, Na^+^, K^+^, NO^3−^, SO_4_^2−^, Cl^−^) and organic compounds (ascorbic acid, glucose).

## References

[1] Khodaei M.M., Alizadeh A., Pakravan N. (2008). Polyfunctional tetrazolic thioethers through electrooxidative/Michael-type sequential reactions of 1,2- and 1,4-dihydroxybenzenes with 1-phenyl-5-mercaptotetrazole. J. Org. Chem..

[2] Zeng Z., Qiu W., Huang Z. (2001). Solid-phase microextraction using fused-silica fibers coated with sol-gel-derived hydroxy-crown ether. Anal. Chem..

[3] Hirakawa K., Oikawa S., Hiraku Y., Hirosawa I., Kawanishi (2002). Catechol and hydroquinone have different redox properties responsible for their differential DNA-damaging ability. S. Chem. Res. Toxicol..

[4] Zhang W., Zheng J., Lin Z., Zhong L., Shi J., Wei C., Zhang H., Hao A., Hu S. (2015). Highly sensitive simultaneous electrochemical determination of hydroquinone, catechol and resorcinol based on carbon dot/reduced graphene oxide composite modified electrodes. Anal. Methods.

[5] Buleandra M., Rabinca A.A., Mihailciuc C., Balan A., Nichita C., Stamatin I., Ciucu A.A. (2014). Screen-printed prussian blue modified electrode for simultaneous detection of hydroquinone and catechol. Sensor. Actuat. B-Chem..

[6] Quan Y., Xue Z., Shi H., Zhou X., Du J., Liu X., Lu X. (2012). A high-performance and simple method for rapid and simultaneous determination of dihydroxybenzene isomers. Analyst.

[7] Li J., Xia J., Zhang F., Wang Z., Liu Q. (2018). An electrochemical sensor based on copper-based metal-organic frameworks-graphene composites for determination of dihydroxybenzene isomers in water. Talanta.

[8] Wang H., Hu Q., Meng Y., Jin Z., Fang Z., Fu Q., Gao W., Xu L., Song Y., Lu F. (2018). Efficient detection of hazardous catechol and hydroquinone with MOF-rGO modified carbon paste electrode. J. Hazard. Mater.

[9] Nagaraja P., Vasantha R.A., Sunitha K.R. (2001). A sensitive and selective spectrophotometric estimation of catechol derivatives in pharmaceutical preparations. Talanta.

[10] Pistonesi M.F., Di Nezio M.S., Centurión M.E., Palomeque M.E., Lista A.G., Fernández Band B.S. (2006). Determination of phenol, resorcinol and hydroquinone in air samples by synchronous fluorescence using partial least-squares (PLS). Talanta.

[11] Ramaraj S., Mani S., Chen S.M., Kokulnathan T., Lou B.S., Ali M.A., Hatamleh A.A., Al-Hemaid F.M.A. (2018). Synthesis and application of bismuth ferrite nanosheets supported functionalized carbon nanofiber for enhanced electrochemical detection of toxic organic compound in water samples. J. Colloid Interface Sci..

[12] Wang H., Zhang S., Li S., Qu J. (2018). Simultaneous determination of hydroquinone and catechol using a glassy carbon electrode modified with Au@Pd loaded on reduced graphene oxide. Anal. Methods.

[13] Deng M., Lin S., Bo X., Guo L. (2017). Simultaneous and sensitive electrochemical detection of dihydroxybenzene isomers with UiO-66 metal-organic framework/mesoporous carbon. Talanta.

[14] Huang Y.H., Chen J.H., Sun X., Su Z.B., Xing H.T., Hu S.R., Weng W., Guo H.X., Wu W.B., He Y.S. (2015). One-pot hydrothermal synthesis carbon nanocages-reduced graphene oxide composites for simultaneous electrochemical detection of catechol and hydroquinone. Sens. Actuat. B Chem..

[15] He J., Qiu F., Xu Q., An J., Qiu R. (2018). A carbon nanofibers-Sm_2_O_3_ nanocomposite: a novel electrochemical platform for simultaneously detecting two isomers of dihydroxybenzene. Anal. Methods.

[16] Zhao L., Yu J., Yue S., Zhang L., Wang Z., Guo P., Liu Q. (2018). Nickel oxide/carbon nanotube nanocomposites prepared by atomic layer deposition for electrochemical sensing of hydroquinone and catechol. J. Electroanal. Chem..

[17] Liu L., Ma Z., Zhu X., Zeng R., Tie S., Nan J. (2016). Electrochemical behavior and simultaneous determination of catechol, resorcinol, and hydroquinone using thermally reduced carbon nano-fragment modified glassy carbon electrode. Anal. Methods.

[18] Li L., Liu D., Mao H., You T. (2017). Multifunctional solid-state electrochemiluminescence sensing platform based on poly(ethylenimine) capped N-doped carbon dots as novel co-reactant. Biosens. Bioelectron..

[19] Jiang H., Wang S., Deng W., Zhang Y., Tan Y., Xie Q., Ma M. (2017). Graphene-like carbon nanosheets as a new electrode material for electrochemical determination of hydroquinone and catechol. Talanta.

[20] Velmurugan M., Karikalan N., Chen S.M., Cheng Y.H., Karuppiah C. (2017). Electrochemical preparation of activated graphene oxide for the simultaneous determination of hydroquinone and catechol. J. Colloid Interface Sci..

[21] Zhang C., Zeng L., Zhu X., Yu C., Zuo X., Xiao X., Nan J. (2013). Electrocatalytic oxidation and simultaneous determination of catechol and hydroquinone at a novel carbon nano-fragment modified glassy carbon electrode. Anal. Methods.

[22] Alshahrani L.A., Liu L., Sathishkumar P., Nan J., Gu F.L. (2018). Copper oxide and carbon nano-fragments modified glassy carbon electrode as selective electrochemical sensor for simultaneous determination of catechol and hydroquinone in real-life water samples. J. Electroanal. Chem..

[23] Shamraiz U., Hussain R.A., Badshah A. (2016). Fabrication and applications of copper sulfide (CuS) nanostructures. J. Solid State Chem..

[24] Roy P., Srivastava S.K. (2006). Hydrothermal growth of CuS nanowires from Cu−Dithiooxamide, a novel single-source precursor. Cryst. Growth Des..

[25] Ke W., Fang G., Lei H., Qin P., Tao H., Zeng W., Wang J., Zhao X. (2014). An efficient and transparent copper sulfide nanosheet film counter electrode for bifacial quantum dot-sensitized solar cells. J. Power Sources.

[26] Zhang X., Wang G., Gu A., Wei Y., Fang B. (2008). CuS nanotubes for ultrasensitive nonenzymatic glucose sensors. Chem. Commun..

[27] Zuo X., Jiao Q., Zhu X., Zhang C., Xiao X., Nan J. (2014). Preparation, characterization and electrochemical properties of a graphene-like carbon nano-fragment material. Electrochim. Acta.

[28] Kundu J., Pradhan D. (2014). Controlled synthesis and catalytic activity of copper sulfide nanostructured assemblies with different morphologies. ACS Appl. Mater. Interfaces.

[29] Venkadesh A., Radhakrishnan S., Mathiyarasu J. (2017). Eco-friendly synthesis and morphology-dependent superior electrocatalytic properties of CuS nanostructures. Electrochim. Acta.

[30] Yang R., Zhang Z., Xu L., Li S., Jiao Y., Zhang H., Chen M. (2017). Laser-induced fabrication of highly branched CuS nanocrystals with excellent near-infrared absorption properties. Chin. Phys. B.

[31] Cabrera-German D., García-Valenzuela J.A., Martínez-Gil M., Suárez-Campos G., Montiel-González Z., Sotelo-Lerma M., Cota-Leal M. (2019). Assessing the chemical state of chemically deposited copper sulfide: A quantitative analysis of the X-ray photoelectron spectra of the amorphous-to-covellite transition phases. Appl. Surf. Sci..

[32] Mattevi C., Eda G., Agnoli S., Miller S., Mkhoyan K.A., Celik O., Mastrogiovanni D., Granozzi G., Garfunkel E., Chhowalla M. (2009). Evolution of electrical, chemical, and structural properties of transparent and conducting chemically derived graphene thin films. Adv. Funct. Mater..

[33] Sun D., Ban R., Zhang P.H., Wu G.H., Zhang J.R., Zhu J.J. (2013). Hair fiber as a precursor for synthesizing of sulfur- and nitrogen-co-doped carbon dots with tunable luminescence properties. Carbon.

[34] Chen Y., Liu X., Zhang S., Yang L., Liu M., Zhang Y., Yao S. (2017). Ultrasensitive and simultaneous detection of hydroquinone, catechol and resorcinol based on the electrochemical co-reduction prepared Au-Pd nanoflower/reduced graphene oxide nanocomposite. Electrochim. Acta.

[35] Wei C., Huang Q., Hu S., Zhang H., Zhang W., Wang Z., Zhu M., Dai P., Huang L. (2014). Simultaneous electrochemical determination of hydroquinone, catechol and resorcinol at Nafion/multi-walled carbon nanotubes/carbon dots/multiwalledcarbon nanotubes modified glassy carbon electrode. Electrochim. Acta.

[36] Zhang Y., Sun R., Luo B., Wang L. (2015). Boron-doped graphene as high-performance electrocatalyst for the simultaneously electrochemical determination of hydroquinone and catechol. Electrochim. Acta.

[37] Huang W., Zhang T., Hu X., Wang Y., Wang J. (2018). Amperometric determination of hydroquinone and catechol using a glassy carbon electrode modified with a porous carbon material doped with an iron species. Microchim. Acta.

[38] Goulart L.A., Mascaro L.H. (2013). GC electrode modified with carbon nanotubes and NiO for the simultaneous determination of bisphenol A, hydroquinone and catechol. Electrochim. Acta.

[39] Gan T., Sun J.Y., Huang K.J., Song L., Li Y.M. (2013). A graphene oxide-mesoporous MnO_2_ nanocomposite modified glassy carbon electrode as a novel and efficient voltammetric sensor for simultaneous determination of hydroquinone and catechol. Sens. Actuat. B Chem..

[40] Liu L., Ma Z., Zhu X., Alshahrani L.A., Tie S., Nan J. (2016). A glassy carbon electrode modified with carbon nano-fragments and bismuth oxide for electrochemical analysis of trace catechol in the presence of high concentrations of hydroquinone. Microchim. Acta.

