# Supplementary material for: 3D-Flower-Like Copper Sulfide Nanoflake-Decorated Carbon Nanofragments-Modified Glassy Carbon Electrodes for Simultaneous Electrocatalytic Sensing of Co-existing Hydroquinone and Catechol
Source: Sensors (Basel). 2019 May 17;19(10):2289. doi: 10.3390/s19102289 (PMC6567201; doi:10.3390/s19102289)
Supplement: Supplementary file 1 [file sensors-19-02289-s001.pdf]

Supplementary information

3D-flower like copper sulfide nanoflakes decorated carbon nanofragments modified glassy carbon electrode for simultaneous electrocatalytic sensing of co-existing hydroquinone and catechol

Lina Abdullah Alshahrani, Liqiong Miao, Yanyu Zhang, Shengming Cheng, Palanivel Sathishkumar, Balasubramaniam Saravanakumar, Junmin Nan, Feng Long Gu*

Key Laboratory of Theoretical Chemistry of Environment, Ministry of Education; School of Chemistry and Environment, South China Normal University, Guangzhou 510006, P. R. China

*__Correspondence:__ gu@scnu.edu.cn (FL Gu)

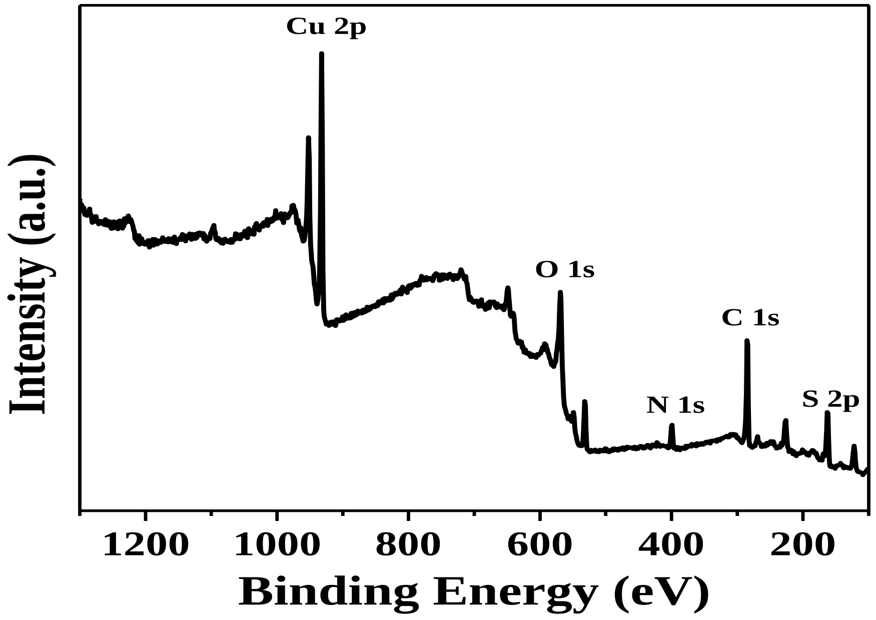

Fig. S1. XPS survey spectrum of CuS nanoflakes anchored CNF.

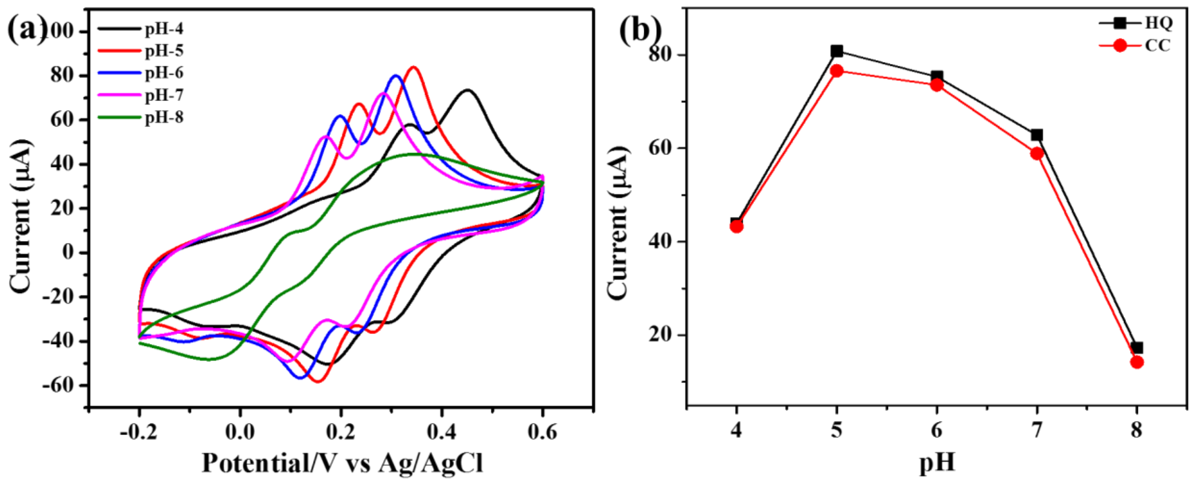

Fig. S2. a) CV curves, b) Current vs pH (DPV curve) of CuS-CNF/GCE in 0.1 M PBS containing 50 μM of HQ and CC at different pH value.

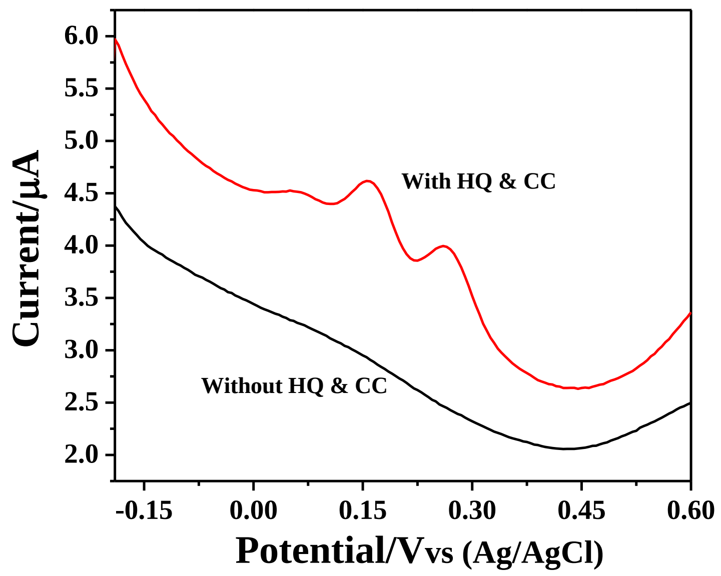

Fig. S3. Interference study of CuS-CNF/GCE in 0.1 M PBS containing 0.5 M of metal ions (Zn^{2+}, Na^{+}, K^{+}, NO^{3-}, SO_4^{2-}, Cl^{-}) and organic compounds (ascorbic acid, glucose) with 50 mM of HQ and CC.”
